# Putting the Evidence into Preceptor Preparation

**DOI:** 10.1155/2012/948593

**Published:** 2012-07-03

**Authors:** Florence Myrick, Florence Luhanga, Diane Billay, Vicki Foley, Olive Yonge

**Affiliations:** ^1^Faculty of Nursing, University of Alberta, Edmonton, AB, Canada T6G 2G3; ^2^University of Regina, Regina, Saskatchewan, Canada S4S 0A2; ^3^First Nations and Inuit Health (FNIH), Health Canada, Edmonton, AB, Canada T5J 4c3; ^4^Faculty of Nursing, University of Prince Edward Island, Canada C1A 4P3

## Abstract

The term evidence-based practice refers to the utilization of knowledge derived from research. Nursing practice, however, is not limited to clinical practice but also encompasses nursing education. It is, therefore, equally important that teaching preparation is derived from evidence also. The purpose of this study was to examine whether an evidence-based approach to preceptor preparation influenced preceptors in a assuming that role. A qualitative method using semistructured interviews was used to collect data. A total of 29 preceptors were interviewed. Constant comparative analysis facilitated examination of the data. Findings indicate that preceptors were afforded an opportunity to participate in a preparatory process that was engaging, enriching, and critically reflective/reflexive. This study has generated empirical evidence that can (a) contribute substantively to effective preceptor preparation, (b) promote best teaching practices in the clinical setting, and (c) enhance the preceptorship experience for nursing students.

## 1. Introduction 

Since the early nineties, a recent development in the health care system and the health professions in general is the prevailing trend towards evidence-based practice, a trend or gold standard against which current practices are being compared [1]. Its ascendancy in the field of research and practice is related to the Cochrane Collaboration in 1993 [2], and also to the efforts of Hargreaves [3] who purported that research in medicine was a model to which educational researchers should aspire. Indeed since that time evidence-based practice (EDP) has become equated with accountable, professional nursing practice [4]. Essentially, the term evidence-based practice refers to the utilization of knowledge, derived primarily from research, in practice. Nursing practice, however, is not restricted to clinical and community-practice but also encompasses the education or teaching of present and future professional nurses. Thus, it is equally important that the teaching practices of nurse educators involved in the different aspects of the educational process are to be also derived from best evidence [5]. According to Stevens and Cassidy [6], evidence-based teaching may be described as “the conscientious, explicit, and judicious use of current best evidence in making decisions about the education of professional nurses” (page 3). 

## 2. Background

The evidence-based preparation of preceptors in this study comprised two full day conferences: (1) preparatory (Day 1); and (2) advanced (Day 2). In the preparatory conference various topics were addressed that were germane to the preceptor's role in the preceptorship experience, for example, preceptor role and responsibilities, the promotion of critical thinking, the process of evaluation, the ethics of teaching in a professional discipline, to name a few. The advanced conference served to augment the substantive content of the preparatory conference and was not merely a repeat of the same content. In the advanced conference such topics as cultural literacy, the intergenerational workplace setting and the use of Benner's model to understand the trajectory of the nurse's professional development were addressed. Additional topics such as teaching learning styles were also addressed. Both conferences were offered onsite/face-to-face in a university setting each over an eight-hour time frame and consisted of didactic as well as interactive sessions. Case study scenarios were interspersed throughout the discussion and dialogue to encourage interaction amongst participants and to generate an environment conducive to critical reflection. In keeping with the research regarding approaches that are most conducive to effective learning, facilitative and engaging learning experiences were thus achieved in these conferences through the use of a variety of teaching and communication processes and that were clearly organized [7–9]. The conferences in this study were designed specifically to facilitate and engage the preceptor participants and to foster a climate that encouraged active engagement and dialogue throughout. The evidentiary material used to inform the discussion was derived from empirical research. As an exemplar, for instance, the session on promoting critical thinking was guided by Myrick's [10] model of enabling critical thinking in preceptorship. According to this research generated model, preceptors promote critical thinking in two ways: (1) *purposively* through their direct questioning of the student; and (2) *incidentally* through their role modelling, guiding, facilitating and prioritizing. In another session entitled “Meeting the Challenges of Precepting an Unsafe Student,” the research findings of [11] were used to inform the discussion around strategies to assist preceptors in dealing with the unsafe student in the clinical setting, while the session on the different generations was derived from a research study by [12]. These empirical works were each first explored didactically with the preceptors. This process was followed immediately by interactive sessions in which the participants explored the scenarios of the different topics amongst themselves. They actively engaged with one another to explore the most appropriate approaches to be pursued to ensure for an effective preceptorship experience. All sessions were thus informed by research and critically examined in that context thereby ensuring that the substantive nature of the conferences was derived from empirical evidence.

## 3. State of Knowledge

While the development of the scholarship of teaching and learning in higher education is important in assisting nurse educators, research is required to evaluate teaching and learning within the context of nursing education [13]. It would be accurate to state that nursing faculty strives consistently to offer educational programs that best reflect teaching practices emanating from evidence in particular research evidence, the implication being that such practice is well informed, current and derives from knowledge that is conducive to the most effective ways to teach in the current health care climate [5]. Such a climate encompasses considerable diversity ranging from cultural to generational to technological advancements that may be described as occurring at a fast pace. In addition, configure into such a climate the academic challenges of increasing class sizes, faculty shortages, decreasing resources, and limited clinical and community teaching sites [5]. To establish a research foundation for nursing education that addresses the contemporary challenges related to nursing education, it is important to develop and evaluate research-based approaches and strategies for nursing education “by enhancing the pedagogic literacy of faculty members (including preceptors)” [13, page 73].

In any professional discipline, the acquisition of knowledge is embodied in its application to the practice setting [14]. Increasingly, nurses must contend with a growing body of knowledge, resolve complex practice issues, adapt to rapid advances in science and technology, and accommodate economic constraints that foster massive health care challenges [15–17]. Indeed, professionals of the future will need to possess, more than ever, the intellectual and emotional capacity for effectively and critically assessing and dealing with the complexity of rapidly changing situations. Consequently, nursing faculty are challenged to provide students with opportunities that will assist them to acquire the competencies necessary to manage the demands of such a dynamic practice. To meet such inexorable demands, it is important that the educators themselves be adequately prepared to teach. Such faculty/teacher/preceptor preparation must in turn derive from teaching practices that derive from the best evidence to support the teaching learning process [18] and Myrick and Yonge [14].

The effect of education on the ability of students to think critically has been the focus of many studies. Outcomes, however, reveal a lack of clarity regarding the mechanisms and operations of the critical thinking process and its applications [19–23]. Preceptorship too has been examined from a variety of perspectives including its impact on the socialization and role transition of students [24–27]; preceptor role modelling [28]; preceptor job satisfaction [29]; and clinical performance of students [30–32]. More recently, preceptorship has been examined to ascertain how it nurtures practical wisdom throughout the learning process of students in the clinical setting [33]. Despite the proliferation of research into preceptorship over the years, findings regarding its impact on clinical teaching, however, continue to remain somewhat inconclusive [34]. 

## 4. Statement of the Problem

Over the last three decades in particular, preceptorship has become the approach of choice for the clinical preparation of undergraduate nursing students. It is a teaching/learning approach, in which students are assigned to expert nurses (preceptors) in the practice setting. This particular arrangement is designed to ensure that students acquire experience on a one-to-one basis with a role model and resource person who is immediately available to them in the practice setting [10, 35] and Myrick and Yonge [14]. To date there has been little structure regarding the preparation of preceptors for the teaching and learning role in the clinical setting. 

## 5. Purpose of the Study

The purpose of this study was to examine an evidence-based approach to prepare preceptors involved in teaching fourth year undergraduate nursing students in the preceptorship experience. The specific objectives of the study were (a) to evaluate the effectiveness of an evidence-based approach to preceptor preparation and (b) to ascertain preceptor perceptions of this preparation in grooming them for their role in the preceptorship experience.

### 5.1. Research Questions

The research questions for this study were as follows: (1) how does the provision of an evidence-based approach contribute to the preparation of preceptors for their role in teaching and learning? (2) Is structured preparation using an evidence-based approach effective in preparing preceptors for their role in the preceptorship experience? (3) How do preceptors perceive their individual approach to the preceptorship experience following such preparation?

## 6. Research Design

### 6.1. Method

A qualitative method using semistructured interviews was used. Constant comparative analysis was used to examine the data. This approach was used to conduct this study because it afforded the researchers the opportunity to deal directly with what was actually going on as to how an evidence-based approach to preceptor preparation influences the teaching practices of preceptors throughout the preceptorship experience. 

### 6.2. Procedures/Data Collection

No less than one month following completion of the workshops, the research assistant carried out data collection during tape-recorded interviews with the participants, which were then transcribed by a professional transcriber. Demographic data were obtained from all participants prior to these interviews. A purposive sample of 29 participants (*n* = 29) was interviewed. Participants were chosen for the study based on the following criteria: they must have been (a) willing to participate in the study; (b) able to speak and understand English; (c) preceptors in the fourth year of the university undergraduate nursing program in which preceptorship was the primary approach to teaching in the practice setting; (d) able to attend one of the structured preceptorship preparatory conferences (full day) provided; and (e) able to sign a consent form agreeing to participate in the study. Preceptors attending the conferences were provided information about the study. A sign-up sheet was made available which they could sign if they were interested in participating. They were also informed that they could withdraw from the study at any time once the process had begun without any fear of reprisal. Interviews were subsequently conducted at a time and place convenient to the participants. A research assistant who had not been connected to the workshops conducted the interviews. An interview guide was used which comprised open-ended questions. Sample questions included “you recently attended a preceptorship workshop, how helpful has it been for you in your preceptor role? What particular area(s) of the workshop have you found most helpful in your role as a preceptor? Has anything about the way you precept students changed following your participation in the workshop? Is there an area/topic you would like to be included in future preceptorship workshops? How would you rate your interest in the preceptorship workshop? Would you recommend this workshop to colleagues and why?” These questions were a beginning guide only and were revised as data emerged. Interviews, each lasting approximately 90 minutes, were conducted with the individual participants in a time and place deemed convenient for them. Secondary data sources included field notes, and personal reflections by the researchers throughout the study. To insure accuracy, data were confirmed by the participants. 

### 6.3. Data Analysis

Constant comparison was used to analyse the data in this study. Intrinsic to that analysis were two levels of coding: (1) substantive and (2) theoretical. Substantive coding involves two levels of coding: open coding and selective coding. Through open coding the data were broken down into discrete components for the purpose of conceptualizing and categorizing the codes [36]. Patterns in the raw data were given conceptual labels through examination of the data line-by-line. As a result of this process, as many codes as possible were generated. The goal was to generate an emergent set of categories and their properties which fit, worked, and were relevant [37]. The second part of substantive coding is selective coding which resulted in the generation of the core variable or the major theme identified in the data. That theme was characterized by the researchers as “Shaping an Evidence-based Pathway to Preceptor Preparation.” At this stage in the analysis, coding was restricted to only those categories which were related specifically to the core variable. When the substantive coding was complete, the second level, also known as theoretical coding, was commenced. At this stage in the analysis the data were put back together in a different way through a process that involved categorizing the data and making links between a category and its substantive codes. The theoretical codes thus conceptualized how the substantive codes related to each other as interrelated hypotheses which in turn accounted for resolving the core variable or theme [38]. 

### 6.4. Mechanisms to Ensure for Rigor

There are four criteria against which rigour in qualitative research is measured [39]. These include credibility, fittingness, auditability, and confirmability. Throughout this study, specific mechanisms were instituted to ensure that these criteria were achieved, thus enhancing the rigour of this investigation. As member checks is the most important mechanism to ensure for rigor in qualitative research, together with mechanisms to ensure for fittingness, auditability and confirmability, credibility was ensured by sharing the findings from the interviews with preceptors to ascertain the accuracy of researcher interpretation.

## 7. Findings and Discussion

Upon examination and ongoing scrutiny of the data, the core variable or theme that emerged in this study was characterized by the researchers as *“*Transforming Preceptorship.” Integral to this transformation were three requisites: (a) engagement; (b) enrichment; and (c) critical reflection/reflexivity. For purposes of discussion and to protect the confidential nature of the study participants, pseudonyms are used throughout the paper to reflect the preceptors' perspectives.

### 7.1. Engagement

According to Palmer [40, page 15] “good teaching is always and essentially communal.” In other words it is important to create a space in which learners can engage in conversation with one another as well as with the subject for which they are convening. This premise was no more apparent than in the process of engagement that surfaced throughout the course of the workshops, a process that was reflected in the preceptors' sentiments. One preceptor, Joan noted:

*“It (the workshop) has been very effective. I really enjoyed it because we got to sit down with other nurses who were experienced, but number two, we got to sit down with students and find out what their ideas have been…I think you learn very much when interacting with both sides. You see the side of the student and then you see your side…The students I was with they were fantastic in voicing what they thought they needed, their experiences and its always helpful to learn both sides.” *

Another preceptor, Mary, stated “you have that flow of information like exchange of information right there, and that's really helpful, really helpful.” Another preceptor, Maureen said, “it was worth hearing other people's experiences as a preceptor and just getting tips from them…I think everyone should do it (attend the workshop) if they're thinking about being a preceptor. Margaret, another preceptor, observed

*“Sitting at the table and talking to the students and being able to hear their concerns, and they were willing to listen too…they are really very worried about the preceptorship and almost felt a bit like it's us against them and I tried to change that us against you or you against us, it is us together for the patient, then that's what our focus should be.”*

Engagement can be considered to be a principle of good teaching and learning, a principle equally important for all kinds of learners [40]. Indeed, of particular importance to the participants in this study was the opportunity to be able to interact with other preceptors and to meet and engage with students in the workshop venue. The opportunity to be able to interact one-to-one and as a collective to discuss and to share experiences with other nurses who themselves were preceptors created a sense of camaraderie amongst the workshop participants that might have otherwise eluded the preceptors had they not attended the workshops. 

Because the students were in attendance, these workshops were also found to provide an excellent medium for the preceptor and the student to interact outside of the clinical environment and more specifically prior to commencement of the preceptorship practicum. Such interaction served to dispel the various myths that may have previously existed about the dyad for both preceptors and students alike. In particular, it afforded preceptors the opportunity to learn firsthand what the students were thinking and feeling about their approaching preceptorship experience and their much anticipated immersion into the world of professional nursing practice. According to Brookfield [41], “having some insight into what students are thinking and feeling…is the foundational, first-order teaching knowledge we need to do good work” (page 28). Such insight in turn can further facilitate and assist the preceptor in his/her approach to the preceptorship experience through an increased understanding of the student's particular perspective. This kind of knowledge can serve to dispel any preconceived ideas on the part of the preceptor and student, diminish or confirm previous held assumptions, and contribute to enhancement of the preceptorship experience.

### 7.2. Enrichment

Participation in the workshop generated for the preceptors the acquisition of new knowledge that ultimately would inform and thus change their perspectives and approach to preceptorship and raise their awareness of the various challenges with which they might be confronted. The evidence-based knowledge that guided and facilitated the discussions and dialogue throughout the workshop served to (a) enhance the preceptors' understanding of the specific responsibilities intrinsic to their preceptor role; (b) increase their individual self-awareness in the preceptor role; (c) promote a greater discernment of their own and the students' expectations; and (d) engender a sense of self-confidence that the preceptor might not have otherwise acquired. As one preceptor, Alison, indicated

*“I had no expectations when I went there (to the workshop). I really didn't know and it just was over and above what I thought it was going to be…I just thought well I'll go and see what new they can tell me after I've been doing this for so long and was wonderfully surprised.” *

Alison went on to describe “the questions that were being asked at the roundtable discussions. Okay the student says this, what would your reactions be and just hearing what the students at my table thought about it.” 

Another preceptor, Tracey, expressed it in the following way:

*“I'm more communicative and not afraid to show them (the students) that I have weaknesses and that I also have strengths, and that they can learn from me but they can also learn from other people on the unit that my way may not be what they feel is the best way but that I'm always also very open to new ideas that they have…Before (the workshop) I might not have communicated that so I think I'm more open about that right off the bat.”*

As one preceptor, Janet, described“I think we pretty much discussed everything that we could and I don't know that there was any topic that we didn't.” Patricia, another preceptor, stated

*“It was to have some clear lines as to what the expectation is and to give you some boundaries as to where are you to go with the student and not overwhelm them with too much and to have a realistic attitude.”*

In the context of this study, the preceptors were provided with the opportunity that emanated from both the knowledge and the dialogue that ensued, to engage in discussion regarding the perception that teaching and learning is a human practice and not simply a repertoire of competencies to be mastered. Teaching and learning was explored and discussed: (a) as a process that is a distinctive way of being human: Hogan [42] affirms this sentiment through the following assertion “teaching in the fullest and most enduring sense of the word, is essentially a commitment to the more worthy fruits of learning itself and a way of being human” (page 29); (b) as being comprised of more than fluency in the skills of teaching; and (c) as being contingent on the relationship that develops between the preceptor and the student which in turn affects the success or lack thereof of the preceptorship experience [14, 42]. One preceptor, Margaret, described, somewhat poignantly, “I think I've been awakened somehow.”

### 7.3. Critically Reflective/Reflexive

“Critical reflection is a hopeful activity [43, page xiii].” According to Myrick and Tamlyn [44], conscious awareness of our own individual teaching practices is essential for establishing a more enlightening experience for both teachers/preceptors and the students alike. While there has been a concerted effort over the past several decades toward the fostering of critical thinking and reflective ability among nursing students, little attention has focused on the ability of nurse educators, including preceptors, to be critically reflective or reflexive about their own approach to teaching. Key to the critical thinking and reflexive process is the ability on the part of the teacher, in this case the preceptor, to be able to identify and scrutinize the assumptions that inform their ideas and actions [41].

An interesting finding in this study was the process of critical reflection and reflexivity that did indeed emerge as a result of the didactic and interactive process that prevailed throughout the workshop sessions. As a consequence of the topics addressed and the dialogue generated preceptors found themselves questioning their own taken-for-granted assumptions. For example, following a session on the intergenerational workplace setting, the current day context in which preceptorship occurs, one preceptor, Judy, stated:

*“The workshop got me saying that the younger generation is all right, they just think differently. And I think that gives me a little bit more of a strength. It makes me look at my student a little bit differently. So I think that's a positive thing…It kind of made me open up a little bit more.”*

As part of this reflexive process, preceptors were found to express a more open perspective toward students of a different generation. Such a process served to influence preceptors in perceiving students as individuals and as learners rather than as being emblematic of a generational cohort with particular characteristics that may or may not be congruent with the preceptor's own generational perspective. 

As with the opportunity for reflexivity, the workshop also engendered a critically reflective dimension. Preceptors found, as a consequence of the discussion and their engagement with the both the topics and the dialogue emanating from them that they began to question their actions and approach to their preceptor role. In other words, they became critically reflective. For example, one preceptor, Ruth, said that as a result of her attendance at the workshop she was “going to question, to think why, why am I doing this or why don't I agree with this, what is it that I am uncomfortable about…you're going to find little questions in our mind that's going to make you dig deeper, go find out something, either from a book or from another person.”

Another preceptor, Sandra, said “I thought the workshop was really, really valuable…assessing your own values and your judgments, working through things with the student rather than just debriefing after something has happened.” Patricia stated, “It (the workshop) has helped me to think critically while Deborah described it in the following way, “It (the workshop) opened my eyes to how difficult it is for the student initially. I have really never thought about that much…I didn't realize that they really come with preconceived ideas.”

## 8. Influence on Preceptors' Approach to Teaching

As a result of participating in the workshop, preceptors indicated that their approach to teaching/precepting was influenced by the knowledge and understanding derived from the didactic and interaction sessions and from the discussions in which they engaged. For example, Nicole, stated “I'm more aware of how they (students) feel…I am more reassuring to them and I value their opinion more as well. Nicole went on further to state

*“Before I would think if I would have a student who wasn't really interested then I would say okay whatever, if you don't want to learn, it's not my problem, but now I think, I'm alone in that. They actually do want to learn and if they get the right approach they do learn and so I see more my responsibility to get them over that step so that they actually learn. I do not believe anymore that they don't want to learn.”*

Insofar as influencing her approach to precepting, one preceptor, Margaret, provided a concrete example. She stated, “I make sure they (the students) are free to ask questions. I will approach them. If I think that they're confused I will introduce some information that will stimulate a question. Another preceptor, Alice, stated “I allow them (students) to think independently and not to do things for them but to say okay you can do this but come to me if you have any questions and then we'll go through it so they're working independently.” Ann stated that, as a result of participating in the workshop, “I'm thinking differently about students…starting really starting with what the students need.”

As can be seen from the preceptors, the workshops contributed toward the way in which they subsequently approached the preceptorship experience. They indicated quite clearly that as a result of their attendance at the workshops, they assumed an informed perspective, one that was derived from the knowledge they had acquired and the discourse that had been generated in the workshop sessions. Their approach to students now emanated from a different stance. For example, rather than make assumptions about student demeanour, they instead now would use the knowledge they had acquired to view the student through a more informed and reflective lens. In other words, they were becoming critically reflective and reflexive. Their approach to the preceptorship experience now became knowledge based, derived from evidence-based teaching and grounded in best practice. 

## 9. Summary and Conclusion

Owing to their participation in these evidence-based workshops, the preceptor participants could be said to have become somewhat transformed through a process of engagement, enrichment, and critical reflection/reflexivity. This transformation was engendered through the acquisition of knowledge and the discussion that ensued with other participants throughout these workshops. Prior to their attendance at these workshops, many of the preceptors had not participated in such a structured preparatory activity. Subsequently, they had embraced the preceptor role in a manner conducive to what they thought to be appropriate, often drawing on their own previous experiences as to how they had been taught or precepted. As a result of the knowledge acquired, the occasion to interact with other nurses who were also assuming the preceptor role and the opportunity to be able to take part in discussions with students and with faculty who facilitated the didactic and interactive sessions, the preceptor participants in this study acquired a new-found understanding of the preceptor role and a more reflective and reflexive understanding of that role. 
